# Knowledge, attitudes, and practices related to infection prevention and control among university dental students in China: a quantitative, questionnaire-based, single-center study

**DOI:** 10.3389/fpubh.2025.1700076

**Published:** 2026-01-13

**Authors:** Wei Lin, Zihe Zhao, Tingyu Liang, Haoxin Pan, Yujie Cao

**Affiliations:** 1 Central Sterile Supply Department, The First Affiliated Hospital, Fujian Medical University, Fuzhou, China; 2 Central Sterile Supply Department, National Regional Medical Center, Binhai Campus of The First Affiliated Hospital, Fujian Medical University, Fuzhou, China; 3 Shanghai Engineering Research Center of Tooth Restoration and Regeneration & Tongji Research Institute of Stomatology & Stomatological Hospital and Dental School, Tongji University, Shanghai, China; 4 Department of Stomatology, The First Affiliated Hospital, Fujian Medical University, Fuzhou, China; 5 Department of Stomatology, National Regional Medical Center, Binhai Campus of The First Affiliated Hospital, Fujian Medical University, Fuzhou, China; 6 Fujian Key Laboratory of Precision Medicine for Cancer, The First Affiliated Hospital, Fujian Medical University, Fuzhou, China

**Keywords:** attitudes, dental students, infection prevention and control, knowledge, practices

## Abstract

**Introduction:**

Dental students may face multiple sources of infection during their clinical practice in the dental environment. In this context, assessing the knowledge, attitudes and practices (KAP) regarding infection prevention and control among dental students is essential. The aim of this study was to conduct a quantitative, questionnaire-based, single-center investigation to assess knowledge, practices, and attitudes of infection prevention and control among university dental students in China.

**Methods:**

A survey was developed and sent to current dental students to assess the KAP regarding infection prevention and control. The questionnaire consisted of 15 items related to knowledge, attitudes, and practices. The reliability of the survey was evaluated using Cronbach’s alpha coefficient (0.67 for knowledge, 0.82 for attitudes, and 0.72 for practice). A one-way analysis of variance (ANOVA) was applied to evaluate the potential differences among groups using the SPSS software. Relationships among scores between KAP were examined using Pearson’s correlation test.

**Results:**

*N* = 218 dental students (response rate: 82.0%) completed the questionnaire. The overall scores of knowledge, attitude, and practice were 2.92 ± 1.07, 3.54 ± 0.54, and 4.03 ± 0.84, respectively. ANOVA indicated significant differences among third-year students, fourth-year students, and interns for attitude (*p* < 0.01) and practice scores (*p* = 0.04), whereas knowledge scores did not differ significantly. Pearson’s correlation test demonstrated the correlation among scores of attitudes and practices (*p* = 0.01). No significant correlations were observed between other parameters.

**Discussion:**

Although dental students in universities generally exhibited positive attitudes and complied with recommended infection prevention and control practices, their knowledge remains insufficient. These results indicate the importance of adding standardized infection control drills in pre-clinical courses and setting up a regular assessment mechanism for infection control knowledge and operational skills to improve clinical training and supervision for dental students in China.

## Introduction

Healthcare-associated infections (HAI) are a health concern globally and are recognized as a leading cause of mortality as well as morbidity related to clinical, diagnostic, and therapeutic procedures (1). HAI constitute a pivotal aspect of iatrogenic harms and represent a critical concern within the framework of patient safety. Transmission of infection between healthcare workers and patients in hospital settings is a particular concern for dental practitioners worldwide. In dental practice, infections may arise from exposure to blood-borne, aerogenic, and droplet-associated pathogens generated during clinical procedures. Transmission may appear through direct contact with secretions or blood. Furthermore, indirect contact with contaminated equipment, droplet spatter, or inhalation of aerosols containing oral fluids may also induce the potential transmission (2, 3).

Infection prevention and control (IPC) refers to strategies implemented by healthcare providers to minimize the potential risk of transmitting infectious agents (4). These measures, including appropriate hand hygiene, strict adherence to dental protocols (specifically covering the sterilization of dental instruments, disinfection of clinical surfaces, management of clinical waste generated during dental procedures), and the application of personal protective equipment (PPE), are formulated based on the primary modes of pathogen transmission (5). Those dentists who fail to implement appropriate infection prevention strategies face not only an elevated risk of acquiring infectious diseases themselves, but also incur the risk of facilitating pathogen transmission between patients in the clinical dental setting. The impact of economic considerations is a critical dimension of IPC, whereby financial incentives for cost reduction may lead dentists to implement inappropriate ICP. Therefore, adherence to safety precautions and established infection-control guidelines is essential.

Currently, several organizations have formulated recommendations for infection control in the dental environment to reduce transmission and promote a safety clinical environment (6). It is also worth emphasizing that interprofessional socialization among diverse healthcare professionals, including physicians, dentists, nurses, and allied healthcare providers, plays a pivotal role in reinforcing the implementation and adherence to standardized infection prevention and control practices in dental care settings. This collaborative interaction can facilitate the consistent sharing of evidence-based infection control knowledge, the alignment of cross-procedural protective protocols, and the timely response to emerging infectious risks across different clinical care scenarios.

Conventional methods for infection prevention include the use of PPE and rigorous hand hygiene. Although these measures are recognized with significant importance, only a small number of clinical practitioners follow all recommended procedures during their clinical practice (7). Procedures in a clinical environment with dental procedures that use spray-generating equipment, such as ultrasonic, rotation instruments, or three-way air or water spray, has been classified as high risk in the dental care environment (8). In terms of the source of the infection, while bacteria are one of the pathogens associated with oral infections, viral pathogens represent the primary occupational risks in dental care settings (9). Because of frequent direct contact with patients and exposure to contaminated instruments, dental students are very vulnerable in the clinical environment (10). There is a strong consensus that enhancing infection prevention and promoting practices among dental students is critical in dental community. Therefore, dental schools must provide sufficient training to students in learning knowledge, cultivating positive attitudes, and ensuring the practice of infection control (11).

The KAP framework describes how professional behavior develops through three interlinked domains. Knowledge, which refers to the information gained through study and experience, plays a key role in forming the basis for informed decisions. Attitudes reflect the dispositions, emotions, and cognitive stance of a person toward tasks or issues. Practice is the observable application of knowledge and norms. In summary, accurate knowledge, positive attitudes, and appropriate practices may guide healthcare providers in delivering patient care (12). However, while the significance of KAP frameworks has been widely emphasized within the dental community, evidence from international settings indicates that dental undergraduates frequently exhibit low compliance with infection control protocols. This finding underscores the critical need to enhance both the foundational knowledge of infection control and the development of positive, practice-aligned attitudes among this population (13). For example, a previous study conducted by Al-Omari found that only 10% of dental students strictly followed infection-control protocols in India (14). Similar investigations in Lebanon and Yemen revealed substantial deficiencies in students’ knowledge and practices in terms of infection control (15, 16). Nevertheless, comprehensive studies specifically examining the KAP regarding infection prevention and control among university dental students in China have yet to be widely documented in the published literature.

Based on the evidence summarized above, the objective of this investigation was to determine the KAP among university dental students in China. In addition, this study also aimed to assess whether significant correlations exist among scores in each domain regarding infection prevention and control.

## Methods

### Study design

The present quantitative, questionnaire-based, single-center study was approved by the Institutional Review Board (Protocol code 2025-SR-[72]). Conducted from July to August 2025, this study involved participants from the School of Stomatology of Tongji University.

### Eligibility criteria and sampling

The convenience sampling approach was used to distribute the questionnaire to 265 students in the School of Stomatology of Tongji University, including third-year students, fourth-year students, and interns, respectively. The inclusion criteria were as follows: (1) aged ≥ 18 years; and (2) currently enrolled as a university student majoring in dentistry. The exclusion criteria were: (1) university students not majoring in dentistry; and (2) a history of mental illness or cognitive impairment that might affect the completion of the study questionnaire or related procedures.

### Study size

According to existing literature, the sample size of this study was calculated using a confidence level of 95%, an expected population proportion of 95.5%, and a margin of error of 3% (11, 17). Specifically, the required sample size (184) was determined on the basis of the following formula:


$$\text{Sample size}\;(n)=Z^{2}\times p\times (1-p)/E^{2}$$

### Data sources and measurement

The survey was developed based on a previously validated tool provided by Singh et al. (11). The self-administered structured questionnaire consisted of 15 closed-ended items regarding KAP related to ICP: six assessing knowledge (Q6, Q7, Q8, Q9, Q10, and Q11), four assessing attitudes (Q3, Q4, Q14, and Q15), and five assessing infection prevention and control practices (Q1, Q2, Q5, Q12, and Q13) (Supplementary material S1). The maximum achievable scores for the knowledge, attitudes, and practices domains were 6, 4, and 5, respectively. For the categorization implementation, raw scores for each participant across the KAP domains were first converted to standardized percentage scores [calculated as: (participant’s raw score/maximum achievable score for the domain) × 100%]. This standardization was performed to eliminate the impact of differing maximum achievable scores across the three KAP domains, ensuring cross-domain comparability of performance. A threshold of 60% was applied to categorize performance in each individual KAP domain as either “adequate” (≥60%) or “inadequate” (<60%). A panel of professional experts conducted a formal evaluation during the Chinese translation process, confirming that the questionnaire’s items accurately reflect the core connotations of IPC KAP and are suitable for Chinese dental students. A formal evaluation of the reliability was conducted using Cronbach’s alpha coefficient (0.67 for knowledge, 0.82 for attitudes, and 0.72 for practice).

### Implementation

All the data were collected online through the Questionnaire Star platform, which is a widely used tool in China. The platform was configured to enforce complete item responses: participants could not submit the questionnaire until all items were answered, thus preventing partial submissions with missing data. Subjects were informed of the objective of the study, the measurement of data confidentiality, and the right to withdraw. All students were explicitly informed that the questionnaire would be completed without collecting personal identifying information. Only those who voluntarily agreed to participate in the research and provided informed consent were permitted to complete the questionnaire.

During data collection, the three groups of students (third-year students, fourth-year students, and interns) completed the questionnaire independently on their personal devices in the classroom via the Questionnaire Star platform, within 15 min and under the supervision of respective researchers. All participants were instructed not to discuss their responses or search for answers online. Additionally, researchers supervised the process to ensure the credibility of the data and addressed any questions raised by students. Importantly, researchers were instructed to only assist with technical issues and avoid engaging with survey content.

### Statistical analysis

In the present study, the collected data were evaluated using SPSS 27.0. Mean and standard deviation were used to express the results of continuous variables. In terms of categorical variables, the results were presented using frequencies and percentages. The Kolmogorov–Smirnov test was applied to evaluate the normality of the data, and the Levene’s test was used to determine the homogeneity of variances. Since the data were normally distributed and met the assumption of equal variances, a one-way analysis of variance (ANOVA) was used to compare the differences in scores among third-year students, fourth-year students, and interns for each variable. The least significant difference test was used as *post hoc* testing to perform further evaluation. In addition, relationships among scores between KAP were examined using Pearson’s correlation test. A *p*-value of ≤ 0.05 was considered statistically significant.

## Results

### Participation metrics

A total of 218 (response rate: 82.0%) of 265 university dental students completed the questionnaire and were included in the study.

### Descriptive results

Participants’ mean age was 23.0 ± 3.28 years. Of the respondents, 52.8% (*n* = 115) were male. The sample comprised 84 third-year students (38.5%), 67 fourth-year students (30.7%), and 67 interns (30.7%) (Table 1).

**Table 1:** Distribution of respondents based on gender and year of study.

Category	Number	Percentage
Gender		
Male	115	52.8
Female	103	47.2
Year of study		
Third-year students	84	38.5
Fourth-year students	67	30.7
Interns	67	30.7

### Main findings

Table 2 summarizes respondents’ KAP regarding infection prevention and control. Most respondents (92.2%) reported washing their hands after and before patient examinations; antiseptic hand solution was the most frequently used method (65.6%). A majority (59.6%) reported using a preprocedural mouth rinse prior to treatment, and all respondents considered patient isolation an important component of infection control. One hundred forty students (64.2%) had received hepatitis B vaccination. Most respondents (*n* = 191, 87.6%) reported using full PPE, including gloves, masks, eye protection, and protective clothing, and 203 (93.1%) disposed of gloves after single use. Nearly all respondents (97.2%) recognized that ineffective sterilization in the clinical dental practice can transmit infections between patients and agreed that, in addition to instrument sterilization, disinfection of dental chairs and clinical areas are needed.

**Table 2:** Knowledge, attitude, and practice of infection prevention and control, by number and percentage of total respondents to each item.

Question	Response	Number	Percentage
Q1. Do you wash your hands before and after patient examination?	Yes	201	92.2
	No	17	7.8
Q2. With what do you wash your hands?	Plain soap	17	7.8
	Detergent	58	26.6
	Antiseptic solution	143	65.6
Q3. Do you prefer oral mouth rinse before commencement of any treatment procedure?	Yes	130	59.6
	No	88	40.4
Q4. Do you think isolation is important in infection control?	Yes	218	100.0
	No	0	0.0
Q5. With which of the following vaccines have you been vaccinated?	Hepatitis B	140	64.2
	Tetanus	24	11.0
	Tuberculosis	6	2.8
	None	48	22.0
Q6. Which of the following do you use to sterilize instruments in dental clinic?	Autoclave	209	95.9
	Boiling	6	2.8
	Washing	3	1.4
Q7. Minimum time required for sterilization in autoclave?	5 min	19	8.7
	10 min	56	25.7
	15 min	143	65.6
Q8. Temperature for sterilization in autoclave?	100 °C	21	9.6
	120 °C	113	51.8
	150 °C	84	38.5
Q9. Which of the following has the highest rate of transmission via saliva?	Hepatitis B	77	35.3
	AIDS	10	4.6
	Tuberculosis	117	53.7
	Do not know	14	6.4
Q10. What immediate action should be taken in case of direct blood contact with an HIV patient?	Anti-HIV immunoglobulins	108	49.5
	Anti-HIV drugs	62	28.4
	Blood tests to be carried out	35	16.1
	Do not know	13	6.0
Q11. Odds of HIV transmission after a single contaminated needlestick injury?	0.1–0.4%	60	27.5
	1–4%	63	28.9
	10–40%	60	27.5
	70–90%	35	16.1
Q12. As a clinician, what protective measures do you take to prevent yourself from injury?	Face mask and gloves	21	9.6
	Eyewear	2	0.9
	Protective clothing	4	1.8
	All the above	191	87.6
Q13. After use of gloves for a patient, what do you do with them?	Dispose of them	203	93.1
	Reuse them after wash	2	0.9
	Reuse them after sterilization	13	6.0
Q14. Ineffective sterilization during clinical practice can transmit infection from one patient to another?	Yes	212	97.2
	No	2	0.9
	Do not know	4	1.8
Q15. Apart from instrument sterilization, disinfection of dental chair, clinic, dental office is required?	Yes	212	97.2
	No	2	0.9
	Do not know	4	1.8

**Figure 1:**
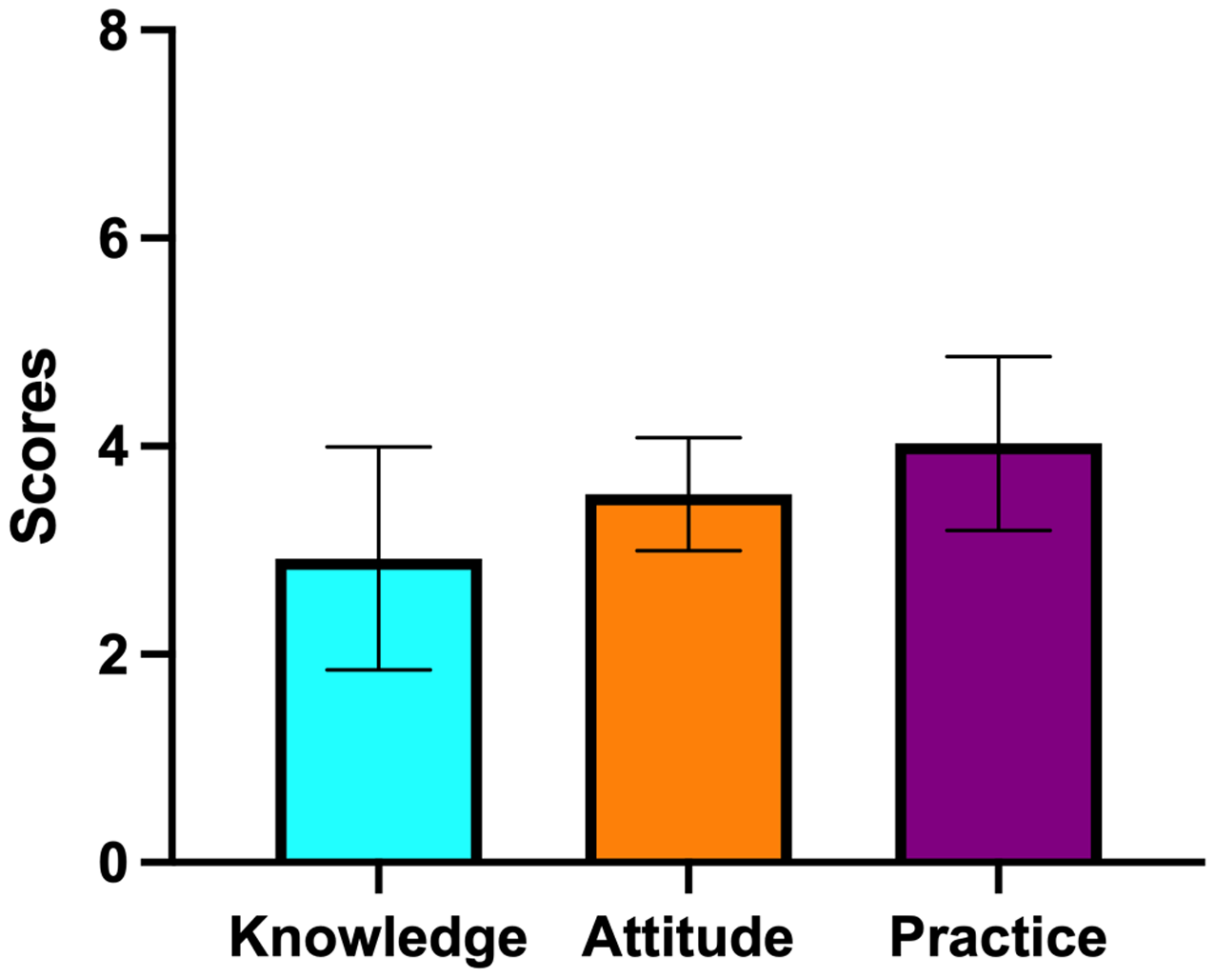
Average scores of knowledge, attitude, and practice regarding infection prevention and control among university dental students.

Mean scores for knowledge, attitudes, and practices were 2.92 ± 1.07, 3.54 ± 0.54, and 4.03 ± 0.84, respectively (Figure 1). A one-way ANOVA indicated significant differences in attitudes (*p* < 0.01) and practices (*p* = 0.04) across academic levels, whereas knowledge scores did not differ significantly (Tables 3, 4). Correlation analysis (Table 5) showed a significant positive correlation between attitudes and practices (p < 0.01). Knowledge showed no correlation with attitudes and practices.

**Table 3:** Knowledge, attitude, and practice scores regarding infection prevention and control among university dental students.

Group	Knowledge	Attitude	Practice
Interns	3.03 ± 1.06	3.36 ± 0.54	3.96 ± 0.77
Fourth-year students	2.76 ± 1.06	3.52 ± 0.51	3.76 ± 0.94
Third-year students	2.91 ± 1.09	3.65 ± 0.53	4.14 ± 0.83
Total	2.92 ± 1.07	3.54 ± 0.54	4.03 ± 0.84
*p*-value	0.48	<0.01	0.04

**Table 4:** Results of least significant difference regarding knowledge, attitudes, and practices between third-year students, fourth-year students, and interns.

Parameter	I	J	I-J	*p*-value
Knowledge	Third-year students	Fourth-year students	0.149	0.48
		interns	−0.123	0.46
	Fourth-year students	interns	−0.272	0.24
Attitudes	Third-year students	Fourth-year students	0.137	0.19
		interns	0.294	<0.01
	Fourth-year students	interns	0.157	0.17
Practices	Third-year students	Fourth-year students	0.386	0.02
		interns	0.189	0.14
	Fourth-year students	interns	−0.198	0.26

**Table 5:** Correlation among knowledge, attitude, and practice scores.

Variables	Correlation coefficient	*p*
Knowledge-attitude	0.03	0.71
Attitude-practice	0.20	<0.01
Knowledge-practice	0.09	0.21

## Discussion

Dental practitioners face an increased risk of blood-borne pathogens because of contact with saliva contaminated with blood, as well as the potential for percutaneous injuries during clinical procedures. Accordingly, following the infection control standards is crucial to minimize the potential risk of transmission and prevent healthcare personnel from infection. Accordingly, this cross-sectional study evaluated the KAP related to infection prevention and control in Chinese university dental students. Overall, the respondents demonstrated positive attitudes and practices toward infection control, but reported relatively poor knowledge. Correlation analysis further showed a positive correction between attitudes and practices, indicating that attitudes are a key predictor of adherence to infection-control measures. To our knowledge, this is the first study in China to evaluate the KAP regarding cross-infection prevention among university dental students.

In recent years, there has been an increased awareness of infectious diseases and the impact of them in clinical practice among various medical disciplines due to the recent COVID-19 pandemic. Although several progress has been made in infection control, there still exist significant gaps that must be addressed in order to promote lifelong adherence to rigorous standards, particularly among dental students (18, 19). For instance, hepatitis B virus is a considerable occupational hazard the healthcare providers who may be exposed to infectious parameters. A previous study conducted in Iran that enrolled 1,628 dental healthcare workers reported the following prevalence results: 0.36% tested positive for hepatitis B surface antigen, 5.0% tested positive for anti-hepatitis B virus core antigen, 0.061% tested positive for anti-hepatitis C virus, and no case was positive for anti-human immunodeficiency virus (20). Hepatitis B vaccination is the most effective method for preventing hepatitis B virus infection. Accordingly, achieving high vaccination coverage among healthcare providers may significantly reduce the transmission of the hepatitis B virus in clinical settings (21, 22). However, only 77 students (35.3%) understood that hepatitis B has the highest transmission rate via saliva. What’s worse, only 140 students (64.2%) in this study had received the hepatitis B vaccine. We assume that this relatively low vaccination rate may be attributed to the absence of a nationwide vaccination policy for healthcare providers in China. There is limited data on vaccination coverage in China and the vaccination rates among healthcare providers in current researches vary ranging from 42.4 to 86.4% (23, 24). Similarly, in terms of HIV prevention and control, only 16.1% of respondents correctly identified the immediate actions when facing direct blood contact with an HIV-positive patient, and merely 27.5% knew the odds of HIV transmission. These findings showed that Chinese dental students have insufficient knowledge in terms of infection prevention. Therefore, it is critical to address these gaps for the purpose of ensuring the safety of the patients as well as minimizing the potential risk of practices among future dental practitioners. Strengthening targeted education on pathogen transmission and mandating HBV vaccination could reduce cross-infection between future dentists and patients in China, especially in resource-limited rural clinics where infection control resources are scarce.

Findings of this study suggest that the current Chinese university dental students exhibit positive attitudes (89%), but their knowledge in this area remains relatively poor (49%). These are consistent with those results from previous researches. For instance, a previous study performed by Srivastava et al. in Saudi Arabia reported that the mean knowledge score of the respondents was 51.6, and 92.1% of participants had a positive attitude (25). Another investigation also indicated a knowledge level of 58.2% among dental students, and 80% of participants reported a positive attitude (26). Accordingly, measures need to be taken to strengthen awareness and implementation of strategies of infection prevention. Integrating scenario-based training into preclinical and clinical curricula could better align student knowledge with practice requirements in China and promote consistency across urban hospitals and grassroots dental facilities. In addition, ANOVA revealed significant differences among the different groups in attitude and practice scores. We interpret this result as potential attitude burnout and increasing laxity in clinical practice over time, highlighting the need for rigorous training of dental students prior to graduation. Furthermore, although practice and attitude were significantly correlated, knowledge showed no correlation with these two parameters. This highlights that cultivating the right attitude is critical for protocol adherence. Accordingly, a comprehensive instruction focusing attitude is essential for shaping behavior in this field.

PPE plays an essential role in protecting healthcare workers from pathogen exposure. Several basic types of PPE, such as masks, gloves or respirators, and gowns, should be easy to get and wear when needed. In our study, most respondents (87.6%) reported that they used full PPE during clinical work, including masks, gloves, eye protection, and protective clothing, which is a promising finding. In addition to PPE, hand hygiene is also necessary for preventing the spread of disease. All clinical staff should follow established protocols of hand hygiene before and after patient contact or sterile procedures. In our sample, 92.2% reported washing their hands before and after patient exams. Hand hygiene is also needed when moving from dirty to clean areas, after patient contact, and after touching contaminated surfaces. Another way is to have patients use an antibacterial mouthwash before treatment. This matches a study from Saudi Arabia: 55% of third- to fifth-year dental students supported this measure, close to the 59.6% in our study (1).

There are several limitations in the present study that need to be emphasized. First of all, the sample size may not comprehensively represent the whole population of dental students in Chinese universities. Dental education in China is characterized by discrepancies in the curricula among different regions and dental institutions, with the majority of dental organizations undergoing the transformation of dental education. Moreover, with the limited questions, the number and scope of the questions may not fully capture respondents’ actual knowledge and practical skills. Criterion validity was not explicitly reported as no gold standard measure was referenced for direct comparison. However, the questionnaire was designed with a minimal number of items to enhance response rates. Another limitation that needs to be considered is the reliance on self-reported data to evaluate adherence to infection control guidelines since the actual clinical practices of respondents could not be observed, responses may not accurately reflect their true KAP. In the future, assessing KAP before and after a health education intervention would have provided stronger evidence in infection prevention and control among dental students. Further research should explore other impact factors such as resource availability, institutional policies, and cultural influences on infection control.

## Conclusion

While university dental students generally demonstrate positive attitudes and comply with recommended IPC practices, their performance on knowledge-related assessments remains suboptimal. These findings highlight the need to strengthen students’ adherence to standardized IPC protocols, as well as to implement formalized, ongoing continuing education initiatives to enhance their related cognitive understanding and knowledge base. The implementation of targeted strategies to improve adherence to evidence-based IPC approaches, alongside the promotion of sustained education and training, could further advance students’ knowledge and awareness in this critical domain. Subsequent supplementary studies may focus on exploring the long-term impacts of scenario-based training modalities, as well as the potential value of personalized training interventions.

## Data Availability

The original contributions presented in the study are included in the article/Supplementary material, further inquiries can be directed to the corresponding author.

## References

[1] Alharbi G Shono N Alballaa L Aloufi A. Knowledge, attitude and compliance of infection control guidelines among dental faculty members and students in KSU. BMC Oral Health. (2019) 19:7. doi: 10.1186/s12903-018-0706-0, 30626370 PMC6325736

[2] Girdler NM Matthews RW Scully C. Use and acceptability of rubber gloves for outpatient dental treatment. J Dent. (1987) 15:209–12. doi: 10.1016/0300-5712(87)90111-4, 3479464

[3] Verrusio AC Neidle EA Nash KD Silverman S Jr Horowitz AM Wagner KS. The dentist and infectious diseases: a national survey of attitudes and behavior. J Am Dent Assoc. (1989) 118:553–62. doi: 10.14219/jada.archive.1989.0082, 2523918

[4] Mangram AJ Horan TC Pearson ML Silver LC Jarvis WR. Guideline for prevention of surgical site infection, 1999. Centers for Disease Control and Prevention (CDC) hospital infection control practices advisory committee. Am J Infect Control. (1999) 27:97–134. doi: 10.1016/S0196-6553(99)70088-X, 10196487

[5] de Souza RA Namen FM Galan J Jr Vieira C Sedano HO. Infection control measures among senior dental students in Rio de Janeiro state, Brazil. J Public Health Dent. (2006) 66:282–4. doi: 10.1111/j.1752-7325.2006.tb04084.x, 17225826

[6] Razak IA Lind OP. Cross-infection control in Malaysian dental practice. Singap Dent J. (1995) 20:11–5. 9582683

[7] Mehtar S Shisana O Mosala T Dunbar R. Infection control practices in public dental care services: findings from one South African Province. J Hosp Infect. (2007) 66:65–70. doi: 10.1016/j.jhin.2007.02.008, 17433494

[8] Melo P Barbosa JM Jardim L Carrilho E Portugal J. COVID-19 Management in Clinical Dental Care. Part I: epidemiology, public health implications, and risk assessment. Int Dent J. (2021) 71:251–62. doi: 10.1016/j.identj.2021.01.015, 33879353 PMC7874946

[9] Rahman B Abraham SB Alsalami AM Alkhaja FE Najem SI. Attitudes and practices of infection control among senior dental students at college of dentistry, university of Sharjah in the United Arab Emirates. Eur J Dent. (2013) 7:S015–9. doi: 10.4103/1305-7456.119058, 24966723 PMC4054074

[10] Al-Maweri SA Tarakji B Shugaa-Addin B Al-Shamiri HM Alaizari NA AlMasri O. Infection control: knowledge and compliance among Saudi undergraduate dental students. GMS Hyg Infect Control. (2015) 10:Doc10. doi: 10.3205/dgkh000253, 26199855 PMC4495767

[11] Singh A Purohit BM Bhambal A Saxena S Singh A Gupta A. Knowledge, attitudes, and practice regarding infection control measures among dental students in Central India. J Dent Educ. (2011) 75:421–7. doi: 10.1002/j.0022-0337.2011.75.3.tb05055.x, 21368266

[12] Jain M Sawla L Mathur A Nihlani T Ayair U Prabu D . Knowledge, attitude and practice towards droplet and airborne isolation precautions amongs dental health care professionals in India. Med Oral Patol Oral Cir Bucal. (2010) 15:e957–61. doi: 10.4317/medoral.15.e957, 20526247

[13] Di Giuseppe G Nobile CG Marinelli P Angelillo IF. A survey of knowledge, attitudes, and behavior of Italian dentists toward immunization. Vaccine. (2007) 25:1669–75. doi: 10.1016/j.vaccine.2006.10.056, 17129642

[14] Al-Omari MA Al-Dwairi ZN. Compliance with infection control programs in private dental clinics in Jordan. J Dent Educ. (2005) 69:693–8. doi: 10.1002/j.0022-0337.2005.69.6.tb03953.x, 15947216

[15] Halboub ES Al-Maweri SA Al-Jamaei AA Tarakji B Al-Soneidar WA. Knowledge, attitudes, and practice of infection control among dental students at Sana'a university, Yemen. J Int Oral Health. (2015) 7:15–9. doi: 10.4103/2231-0762.156152, 26028896 PMC4441229

[16] Dagher J Sfeir C Abdallah A Majzoub Z. Infection control measures in private dental clinics in Lebanon. Int J Dent. (2017) 2017:1–11. doi: 10.1155/2017/5057248, 28642792 PMC5470049

[17] Qamar MK Shaikh BT Afzal A. What do the dental students know about infection control? A cross-sectional study in a teaching hospital, Rawalpindi, Pakistan. Biomed Res Int. (2020) 2020:3413087. doi: 10.1155/2020/3413087, 32596299 PMC7285392

[18] Laheij AM Kistler JO Belibasakis GN Välimaa H de Soet JJ European Oral Microbiology Workshop (EOMW) 2011. Healthcare-associated viral and bacterial infections in dentistry. J Oral Microbiol. (2012) 4:10. doi: 10.3402/jom.v4i0.17659 PMC3375115 22701774

[19] Ayatollahi J Ayatollahi F Ardekani AM Bahrololoomi R Ayatollahi J Ayatollahi A . Occupational hazards to dental staff. Dent Res J (Isfahan). (2012) 9:2–7. doi: 10.4103/1735-3327.92919, 22363355 PMC3283973

[20] Ahmad Akhoundi MS Momeni N Norouzi M Ghalichi L Shamshiri AR Alavian SM . Prevalence of blood-borne viruses among Iranian dentists: results of a national survey. Int J Occup Med Environ Health. (2015) 28:593–602. doi: 10.13075/ijomeh.1896.00324, 26190734

[21] Zheng YB Gu YR Zhang M Wang K Huang ZL Lin CS . Health care workers in Pearl River Delta area of China are not vaccinated adequately against hepatitis B: a retrospective cohort study. BMC Infect Dis. (2015) 15:542. doi: 10.1186/s12879-015-1278-0, 26590815 PMC4655081

[22] Magnavita N Puro V. Management of HBV infected health care workers. J Clin Virol. (2003) 27:310–1. doi: 10.1016/S1386-6532(03)00126-4 12878096

[23] Smithers P Murray SB Stewart S Skull S. Hospital health care worker (HCW) vaccination coverage after implementation of an HCW vaccination policy. Aust Health Rev. (2003) 26:76–83. doi: 10.1071/AH030076, 15485377

[24] Yuan Q Wang F Zheng H Zhang G Miao N Sun X . Hepatitis B vaccination coverage among health care workers in China. PLoS One. (2019) 14:e0216598. doi: 10.1371/journal.pone.0216598, 31063488 PMC6504080

[25] Srivastava KC Shrivastava D Sghaireen MG Alsharari AF Alduraywish AA al-Johani K . Knowledge, attitudes and practices regarding COVID-19 among dental health care professionals: a cross-sectional study in Saudi Arabia. J Int Med Res. (2020) 48:300060520977593. doi: 10.1177/0300060520977593, 33307897 PMC7739093

[26] Shrivastava D Alduraywish AA Srivastava KC Alsharari AF al-Johani K Sghaireen MG . Assessment of knowledge and attitude of allied healthcare professionals about COVID-19 across Saudi Arabia. Work. (2021) 68:305–15. doi: 10.3233/WOR-203377, 33492261

