# Supplementary material for: Knowledge, attitudes, and practices related to infection prevention and control among university dental students in China: a quantitative, questionnaire-based, single-center study
Source: Front Public Health. 2026 Jan 13;13:1700076. doi: 10.3389/fpubh.2025.1700076 (PMC12834792; doi:10.3389/fpubh.2025.1700076)
Supplement: Supplementary file 1 [file Table_1.DOCX]

**Questionnaire**

**1. General questions:**

1)Please indicate your age: _____

2)Please indicate your gender: □Male □Female

3)Please indicate which year of study you are in: □3rd □4th □Intern

**2.Knowledge, attitudes, and practices of infection prevention and control**

Q1. Do you wash your hands before and after patient examination? □Yes □No

Q2. With what do you wash your hands? □Plain soap □Detergent □Antiseptic solution

Q3. Do you prefer oral mouth rinse before commencement of any treatment procedure? □Yes □No

Q4. Do you think isolation is important in infection control? □Yes □No

Q5. With which of the following vaccines have you been vaccinated?

□Hepatitis B □Tetanus □Tuberculosis □None

Q6. Which of the following do you use to sterilize instruments in dental clinic?

□Autoclave □Boiling □Washing

Q7. Minimum time required for sterilization in autoclave? □5min □10min □15min

Q8. Temperature for sterilization in autoclave? □100°C □120°C □150°C

Q9. Which of the following has the highest rate of transmission via saliva?

□Hepatitis B □AIDS □Tuberculosis □Don’t know

Q10. What immediate action should be taken in case of direct blood contact with an HIV patient? □Anti-HIV immunoglobulins □Anti-HIV drugs □Blood tests to be carried out □Don’t know

Q11. Odds of HIV transmission after a single contaminated needlestick injury?

□0.1%–0.4% □1%–4% □10%–40% □70%–90%

Q12. As a clinician, what protective measures do you take to prevent yourself from injury? □Face mask and gloves □Eyewear □Protective clothing □All the above

Q13. After use of gloves for a patient, what do you do with them?

□Dispose of them □Reuse them after wash □Reuse them after sterilization

Q14. Ineffective sterilization during clinical practice can transmit infection from one patient to another? □Yes □No □Don’t know

Q15. Apart from instrument sterilization, disinfection of dental chair, clinic, dental office is required? □Yes □No □Don’t know

**Annotation**

For each question, if the content of the answer matches the following options, it will be awarded 1 point; all other options will be awarded 0 points.

Q1:Yes

Q2:Antiseptic solution

Q3:Yes

Q4:Yes

Q5:Hepatitis B

Q6:Autoclave

Q7:15min

Q8:120°C

Q9:Hepatitis B

Q10:Blood tests to be carried out

Q11:0.1%–0.4%

Q12:All the above

Q13:Dispose of them

Q14:Yes

Q15:Yes
